# A performance evaluation of despiking algorithms for eddy covariance data

**DOI:** 10.1038/s41598-021-91002-y

**Published:** 2021-06-02

**Authors:** Domenico Vitale

**Affiliations:** 1 Department for Innovation in Biological, Agro-food and Forest systems (DIBAF), University of Tuscia, via San Camillo de Lellis, 01100 Viterbo, Italy; 2 Euro-Mediterranean Centre on Climate Change Foundation (CMCC), Lecce, 73100 Italy

**Keywords:** Ecology, Ecological modelling, Biogeochemistry, Carbon cycle, Mathematics and computing, Computational science, Statistics

## Abstract

Spike detection for raw high-frequency eddy covariance time series is a challenging task because of the confounding effect caused by complex dynamics and the high level of noise affecting such data. To cope with these features, a new despiking procedure rooted on robust functionals is proposed. By processing simulated data, it is demonstrated that the proposed procedure performs better than the existing algorithms and can be therefore considered as a candidate for the implementation in data center environmental monitoring systems, where the availability of automatic procedures ensuring a high quality standard of released products constitutes an essential prerequisite.

## Introduction

Quantifying greenhouse gases (GHGs) emitted to and removed from the atmosphere is fundamental for the development of mitigation strategies in response to climatic changes. To this end, an important research frontier in ecology is directed toward measuring the rates of exchange (or flux) of GHGs over natural ecosystems and agricultural fields^1–3^.

Surface layer fluxes of water (H$_2$O), carbon dioxide (CO$_2$), methane (CH$_4$) and nitrous oxide (N$_2$O) are nowadays calculated by using the eddy covariance (EC) technique^4^. This technique employs a sonic anemometer for wind velocity components and a gas analyzer for scalar atmospheric concentrations and involves high-frequency sampling (at least 10 observations per second). Half-hourly eddy fluxes are derived from the covariance (normally within an averaging time of 30 min) between high-frequency time series of vertical wind speed and atmospheric concentration of the scalar variable of interest, which can be temperature, water vapor, carbon dioxide or any other trace gas, measured at the same point in space and time.

Despite improvements in measurement instruments and data acquisition systems, rigorous quality control (QC) routines are required to identify and remove any flux values affected by significant bias^5^. An important part of QC routines deals with the identification of unexpectedly impulsive peaks (spikes) in raw data involved in flux covariance estimation. Water on the sonic anemometer transducers, artifacts due to insects, faulty of the power supply, data transmission errors, to name a few, represent typical events generating spikes in raw EC data^6,7^.

The presence of spikes in raw data may introduce significant bias in derived fluxes. Beyond the magnitude of the spikes, as well as on their amount, the bias introduced in flux estimates has a different size depending on whether the presence of anomalous spikes affect just one of the two times series necessary for covariance calculations, or both simultaneously^8^. The presence of spikes can also have disturbing effects on the cross-covariance function (which is extensively used to align time series sampled by different measurement instruments), on higher-order moment estimates (such as variance and kurtosis), as well as on OLS regression coefficients (used for linear trend removal) and (co)spectral density (in this case, spikes pose a problem for the estimation of reliable spectral correction factors used to adjust for flux attenuation due to sensors separation).

Spike detection for raw, high-frequency, EC data is a challenging task because the complex time series dynamics and the high level of noise makes it difficult to distinguish between good and spiky data points. At the same time, with high dimensional data of this kind (in case of data sampled at 10 Hz scanning frequency and stored in files of 30-min interval, each time series has 18,000 observations), robust, unsupervised and computationally efficient algorithms are of great interest, in particular for data center environmental monitoring systems, where ensuring that the key functions and all services are delivered without any interruptions or abnormalities constitutes an essential prerequisite.

A variety of procedures (described in more detail in the "Methods" section) has been proposed for the detection and removal of spikes. However, there is no general agreement as to which method is the best, because the performance of despiking algorithms for EC data is generally unknown. Recently, Starkenburg et al.^9^ addressed the problem of performance evaluation of despiking algorithms for EC data by means of a simulation study. However, they did not consider the possible presence of heteroskedastic dynamics in time series which is an important feature of raw, high-frequency EC data.

In the light of these considerations, this paper has a twofold purpose. First, we propose a new despiking procedure for EC data based on robust functionals. The procedure involves *i*) a preliminary signal extraction, followed by *ii*) an outlier score computation for each observed data point. To this end, we considered a robust regression technique based on repeated median filtering^10^, which has been widely demonstrated to perform better than such standard filtering methods as running median when time series are characterized by local temporal trends dynamics^11^. For the scale parameter of the residual component, the $Q_n$ estimator^12^ was selected because of its suitability to prevent implosion of the estimates when data are characterized by extreme low variability^13^, as often encountered in real, observed EC data^5^. Both the functionals were calculated in a sliding window of moderate size, to account for the complex dynamics of the signal and the heteroskedastic behaviour of the noise component.

Second, we carry out a Monte Carlo experiment aiming at comparing the ability of the existing despiking algorithms with the newly proposed one. To this end, we simulated data from an autoregressive integrated moving average (ARIMA) process with heteroskedastic error structure, in such a way to mimic the stochastic properties of raw, high-frequency, EC time series as closely as possible. Subsequently, simulated data were contaminated by a suitable amount of spikes with varying magnitudes as commonly encountered in real, observed data, to quantitatively assess the merits and demerits of each despiking algorithm.

## Methods

### A review of existing despiking procedures

Among despiking algorithms for raw, high-frequency, EC data, a popular approach was developed by Vickers and Mahrt^6^ (hereinafter VM97). The method consists in estimating the sample mean and standard deviation in overlapping temporal windows whose width in time is 5 min. The temporal window slides point by point, and any data point whose value exceeds $\pm 3.5 \sigma$ (sample standard deviation) is flagged as a spike. The method is highly sensitive to the masking effect (where less extreme spikes go undetected because of the existence of the most extreme spikes), a reason for which the procedure is iterated increasing by 0.1 the threshold value at each pass, until no more spikes are detected.

A revised version of the VM97 procedure was proposed by Metzger et al.^14^ (hereinafter M12), who suggested replacing the mean and standard deviation by more robust estimates, such as the median and the median absolute deviation (MAD), respectively. The authors found that this method reliably removed spikes that were not detected by VM97, showing a superior performance.

To reduce the high-computational burden attributable to the windowed computations prescribed by the VM97 algorithm, Mauder et al.^7^ (hereinafter M13) proposed to estimate median and MAD over the whole flux averaging period (usually 30 or 60 min). M13 suggested to consider as spike those observations exceeding $\pm 7\cdot$MAD. Such an approach was selected as candidate method in the data processing scheme at the ICOS ecosystem stations^15^.

Starkenburg et al^9^ recommended the approach developed by Brock^16^ (hereinafter BR86) as the best method for despiking EC data. This algorithm is currently implemented in the processing pipeline adopted by the National Ecological Observatory Network (NEON, https://www.neonscience.org). It is based on a two-stage procedure, where the first step consists in extracting the signal by means of a rolling third-order median filter which replaces the center value in the window with the median value of all the points within the window; the second step aims at identifying spikes by analyzing the histogram of the differences between the raw signal and the median filtered signal. Specifically, the differences are initially binned into 25 classes. Then, the first bins with zero counts on either side of the histogram are identified and points in the original signal that exceed the empty bins are flagged as spikes. If no bin with zero counts is found, then the number of bins is doubled (for example from 25 to 51, with one bin added ensuring to retain an odd number because the mean of differences, which is expected to be close to zero, should fall into the central bin of the histogram). The procedure is iterated by increasing the number of bins until the bin width is not less than the acquiring instrument resolution.

### The proposed despiking algorithm

**Figure 1:**
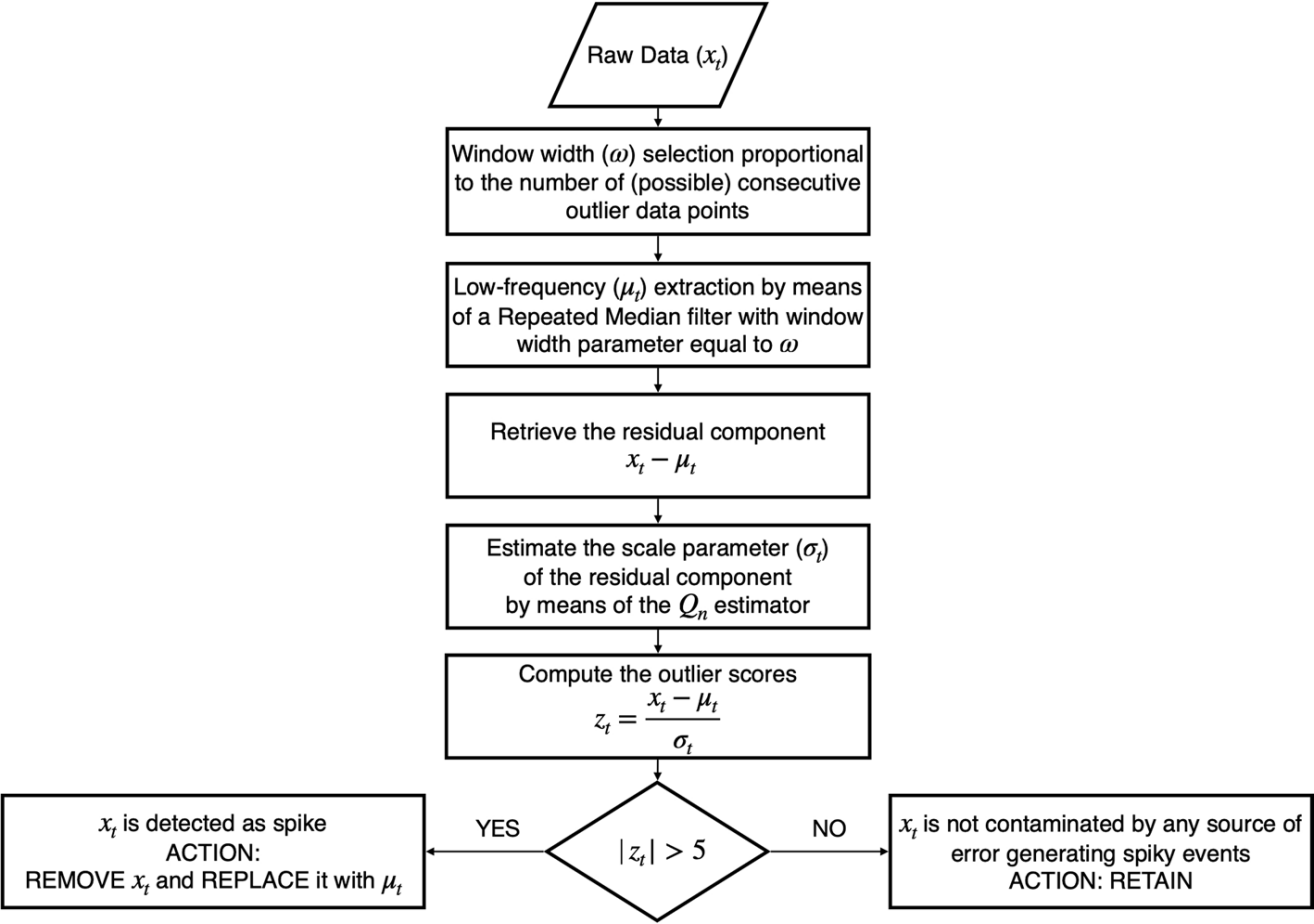
Flowchart of the proposed despiking algorithm.

In order to define a modeling framework suitable for the representation of a sequence $(x_t)_{t \in Z}$ of observed raw EC data indexed by time *t* and contaminated by spikes, we assume a component model as follows:

1 $$\begin{aligned} \left. \begin{aligned} x_t&= \mu _t + v_t + s_t,\\ \end{aligned}\right. \end{aligned}$$

where $\mu _t$ denotes the low frequency component (signal); $v_t$ the deviations from the signal level (residuals) whose variability ($\sigma _t^2$) is allowed to change slowly over time; and $s_t$ the spike generating mechanism which is zero most of time but occasionally generates large absolute values.

To achieve unbiased estimates of both the signal and the scale parameter ($\sigma _t$) when data are contaminated by errors, the use of robust estimators is required. One of the most popular measures of robustness of a statistical procedure is the *breakdown point*, which represents the proportion of outlying data points an estimator can resist before giving a biased result. The maximum breakdown point is 50%, since, if more than half of the observations are contaminated, it is not possible to distinguish between the distribution of good data and the distribution of outlying data. Described in these terms, the arithmetic mean has a breakdown point of 0% (i.e. we can make the mean arbitrarily large just by changing any of the data point), whereas the median has a breakdown point of 50% (i.e. it becomes biased only when 50% or more of the data are large outliers).

The proposed despiking procedure (hereinafter RobF) makes use of robust functionals whose breakdown point is 50% and consists in three stages (see Fig. 1). In the first step the signal ($\mu _t$) extraction is carried out by means of the repeated median (RM) regression technique^10,17^. The second step involves the estimation of the time-varying scale parameter $\sigma _t$ by means of the $Q_n$ estimator^12^. A detailed description of the robust functionals will be provided in the following sections. Spikes are detected in the third step, through the examination of outlier scores calculated as:

2 $$\begin{aligned} z_t=\frac{x_t-\mu _t}{\sigma _t}. \end{aligned}$$

Any values of $|z_t|$ exceeding a pre-fixed threshold value ($z_{th}$) is considered as spike. The choice of the threshold value should be based on the outlier scores data distribution which can vary across time. In this work $z_{th}$ was set equal to 5 which means that for Normal- and Laplace-distributed data there is a 1 in 3.5 million and 1 in 300 chance, respectively, that an anomalous value is the result of a statistical fluctuation over the spectrum of plausible values. Once detected, spikes are removed and replaced by $\mu _t$ estimates obtained by the RM filter.

#### Repeated median filter

The idea underlying moving time window based approaches is that of approximating the signal underlying observed data by means of local estimates that approximate the level of data in the center of the window.

To this end, we fit a local linear trend^11^ of the form

3 $$\begin{aligned} \mu _{t+i}=\mu _t+i\beta _t, \quad i=-k,\ldots ,k, \quad \mathrm {to} \quad \{x_{t-k},\ldots ,x_{t+k}\}, \end{aligned}$$

where *k* is the parameter defining the time window of length $n=2k+1$, whereas $\mu _t$ and $\beta _t$ are estimated by means of the RM filter^10^ as

4 $$\begin{aligned} \left. \begin{aligned} \tilde{\mu }_t^{RM}&=med\bigl (x_{t-k}+k\tilde{\beta }_t,\ldots ,x_{t+k}-k\tilde{\beta }_t\bigr ),\\ \tilde{\beta }_t^{RM}&=med_{i=-k,\ldots ,k} \Bigl (med_{j=-k,\ldots k,j \ne i} \frac{x_{t+i}-x_{t+j}}{i-j}\Bigr ). \end{aligned}\right. \end{aligned}$$

The only parameter required for the application of the RM filter is *k*, which controls how many neighbouring points are included in the estimation of $\mu _t$. Its choice depends not only on the time series characteristics, but also on the situations a procedure needs to handle. For despiking purposes, *k* has to be chosen as a trade-off problem between the duration of periods in which trends can be assumed to be approximately linear and the maximum number of consecutive outliers the estimator allows to resist before returning biased results.

Results of previous studies^18^ for the evaluation of the RM filter performance in the removal of patches of impulsive noise showed that the RM resists up to 30% subsequent outliers without being substantially affected. Therefore, the minimal window width should be larger than at least three times the maximal length of outlier patches to be removed.

To this end, the optimal time window width selection is carried out through a preliminary analysis of the data distribution. Specifically, the time series is subject to a preliminary de-trending procedure, where trend is approximated by a 5-degree polynomial function whose parameters are estimated via iterated re-weighted least squares (IWLS) regression. The optimal window width is then set equal to 4 times the maximum number of values exceeding $\pm 3\cdot s_g$ in 30 s intervals, where $s_g$ is the (global) standard deviation estimated by the $Q_n$ estimator on de-trended data. To prevent cases where few or no data exceed the threshold values, a minimum window width of 5 s is imposed (i.e. 51 time steps for data sampled at 10 Hz acquisition frequency).

#### ${{Q}}_n$ scale estimator

Beyond the ability of the filter adopted for signal extraction, the effectiveness of a despiking strategy depends also on the robustness of the scale parameter, $\sigma _t$, which is of fundamental importance for the outlier scores derivation. Raw EC time series cannot be assumed to be identically distributed as variability may vary over time as the effect of changes in turbulence regimes and heterogeneity of the flux footprint area. In such situations, global estimates of the scale parameter are unrepresentative of the local variability. Consequently, the spike detection procedure becomes ineffective. To cope with this feature, the scale parameter $\sigma _t$ was estimated in rolling time windows whose width was set equal to those adopted for the signal extraction. As a robust estimates of $\sigma _t$, we used the $Q_n$ estimator^12^

5 $$\begin{aligned} Q_n=2.2219\{|x_i-x_j|;i<j\}_{(q)}, \end{aligned}$$

with $q=$ ${h}\atopwithdelims (){2}$ $\approx$ ${n}\atopwithdelims (){2}$ $/4$ and $h=\lfloor \frac{n}{2} \rfloor +1$, where $\lfloor \cdots \rfloor$rounds down to the nearest integer^19^. This scale estimator corresponds approximately to the first quartile of all pairwise distances between any two data points. Compared to other robust scale estimators - such as the MAD and the interquartile distance IQD - it is a location-free estimator, i.e. it does not implicitly rely on a symmetric distribution. However, its efficiency is larger especially when identical measurements occur, e.g. due to limited resolution of the measurement instruments^13^.

#### Software implementation

The application of the robust estimators previously described is hampered because of their high computational demands^20^. For example, the slope $\tilde{\beta }_t^{RM}$ within the time window is estimated by taking repeated medians of pairwise slopes, namely an inner median with one observation being fixed, and then the median of all these inner medians. As a consequence, when applied to large time windows the RM estimator may require high intensity calculation. To cope with this problem, we used the algorithm developed by Bernholt et al.^17^ and implemented in the R package robfilter^21^ which allows to update the RM filter by using the stored information from the last time window. Since estimates do not have to be calculated for each window from scratch, the computational demand is significantly reduced. For $Q_n$ estimator, the implementation available in the R package robustbase^22^ was used.

To further reduce the computational time, the proposed despiking procedure was run in parallel mode. The parallelization consists in the simultaneously processing of 5 min length time series (provided the window width is less than 1 min). Such an improvement makes the computation of the both RM filter and $Q_n$ scale estimator much faster and then suitable for the analysis of such high-dimensional data as the raw EC time series.

### Monte Carlo experiment

#### Simulation design

Performance evaluation of despiking algorithms is a difficult task because the true labels (good data/spike) of individual data points are not always available. To overcome such limitations, we carried out a Monte Carlo simulation study. Simulations allow, in fact, to get a full control of the spike-generating mechanism and, consequently, a more objective performance evaluation of the despiking algorithms becomes feasible.

To mimic stochastic properties of raw, high-frequency EC data as closely as possible, synthetic time series were simulated from autoregressive integrated moving average ARIMA(p,d,q) processes with a time-varying error structure, the latter generated by means of the component generalized autoregressive heteroskedastic (CGARCH) model^23^. The ARIMA modeling framework aims at simulating the conditional mean process, whereas the CGARCH model aims at mimicking the non-constant conditional variance process.

The basic form of an ARIMA(p,d,q) process can be written as:

6 $$\begin{aligned} \nabla ^d X_t = c + \sum _{i=1}^p \phi _i \nabla ^d X_{t-i} + \sum _{j=0}^q \theta _j \varepsilon _{t-j}, \end{aligned}$$

where *p* is the order of the AR part and *q* is the order of the MA part, $\nabla ^d$ denoting the difference operator of order *d*. The process $X_t$ is stationary if and only if $d=0$, in which case it reduces to an ARMA(p,q) process. If $d>0$, then the process $X_t$ is said to be integrated of order *d*, meaning that $X_t$ needs to be differenced *d* times to achieve stationarity. To allow heteroskedasticity, we assume that $\varepsilon _t= \sigma _t e_t$, where $e_t$ is a sequence of independently and identically distributed variables with mean 0 and variance 1 and $\sigma _t^2$ is the conditional variance allowed to vary with time.

The latter was simulated by means of a CGARCH process, which can be written as:

7 $$\begin{aligned} \left. \begin{aligned} \sigma _t^2&=q_t + \sum _{i=1}^r \alpha _i (\varepsilon _{t-i}^2 - q_{t-i}) + \sum _{j=1}^s \beta _j (\sigma _{t-j}^2 -q_{t-j})\\ q_t&=\omega + \eta _{11} q_{t-1} + \eta _{21} (\varepsilon _{t-1}^2 - \sigma _{t-1}^2), \end{aligned}\right. \end{aligned}$$

where $\omega$, $\alpha _i$, $\beta _j$, $\eta _{11}$, $\eta _{21}$ are strictly positive coefficients; $q_t$ is the permanent (long-run) component of the conditional variance allowed to vary with time following first order autoregressive type dynamics. The difference between the conditional variance and its trend, $\sigma _{t}^2 - q_{t}$, is the transitory (short-run) component of the conditional variance. The conditions for the non-negativity estimation of the conditional variance^23^ are related to the stationary conditions that $\alpha _i + \beta _j < 1$ and that $\eta _{11} < 1$ (such quantities provide a measure of the persistence of the transitory and permanent components, respectively).

Model order specification and parameter estimation were performed by analyzing real EC data (more detail are provided in the “Results and discussion” section). With this modelling framework, we simulated 18,000 values as in EC raw data sampled at 10 Hz scanning frequency within a 30-min interval. Simulations were executed in the R v.4.0.2 programming environment by using the tools implemented in the rugarch package^24^.

Once simulated, synthetic time series were intentionally corrupted with 180 spiky data points (1% for a sample size of 18000). Two macro-scenarios were considered. In the first scenario (S1), isolated or consecutive spike events of short duration were generated. In particular, 180 spike locations were randomly selected in such a way to obtain 30 single spikes, 30 spikes as double (consecutive) events, and 30 spikes as triple (consecutive) events. In the second scenario (S2), instead, time series were contaminated by impulsive peaks of longer duration. To this end, spike locations were carried out by randomly selecting five blocks of 50 consecutive data points. Once located, spikes were generated by multiplying the corresponding time series values (after mean removal) for a factor 10 in such a way to have magnitude similar to those commonly encountered on real, observed EC data. To simulate consecutive spike events as imposed by S2 scenario, generated spiky data points were taken in absolute term. Each scenario was permuted 99 times.

#### Metrics

The ability of the despiking algorithms was assessed by comparing the number of artificial spikes inserted into the time series with the number of spikes identified by the method. More particularly, by referring to the $2\times 2$ confusion matrix as reported in Table 1, a valid despiking procedure maximizes decisions of type *true positive* (TP) while, at the same time, keeping decisions of the types *false negative* (FN) and *false positive* (FP) at the lowest levels possible. This trade-off can be measured in terms of *Precision* and *Recall*, which are commonly used for measuring the effectiveness of set-based retrieval^25^. For any given threshold value, the Precision is defined as the fraction of reported spikes that truly turn out to be spikes:

8 $$\begin{aligned} \text {Precision}=\frac{\text {TP}}{\text {TP}+\text {FP}}, \end{aligned}$$

while the Recall is correspondingly defined as the fraction of ground-truth spikes that have been reported as spikes:

9 $$\begin{aligned} \text {Recall}=\frac{\text {TP}}{\text {TP}+\text {FN}}. \end{aligned}$$

**Table 1:** Confusion matrix.

	Detected as spike	Not detected as spike
True spike	True positive (TP)	False negative (FN)
Good data	False positive (FP)	True negative (TN)

As a measure that combines Precision and Recall, we consider the balanced F1-Score, which is the harmonic mean of the two indices above-mentioned, and given by:

10 $$\begin{aligned} \text {F1-Score}=2 \cdot \frac{\text {Precision} \cdot \text {Recall}}{\text {Precision} + \text {Recall}}. \end{aligned}$$

We have $0\le \text {F1-Score} \le 1$ where 0 implies that no spikes are detected and 1 indicates that all, and only, the spikes are detected. The closer to 1 the F1-Score index, the greater the effectiveness of the despiking method.

In addition to the previous outlined metrics, a comparison between variances of (simulated) uncorrupted time series and the one estimated after the application of the despiking procedure has been performed.

For an overall evaluation of the performance of the despiking algorithms, the Friedman test^26^ using a significance level $\alpha =0.05$, followed by a post-hoc test based on the procedure introduced in Nemenyi^27^ was applied. The Friedman test is a non-parametric statistical test, equivalent to repeated-measures ANOVA, which can be used to compare the performances of several algorithms^28^. The null hypothesis of the Friedman test is that there are no significant differences between performances of all the considered algorithms. Provided that significant differences were detected by the Friedman test (that is the null hypothesis is rejected) the Nemenyi test can be used for pairwise multiple comparisons of the considered algorithms. Nemenyi test is similar to the post-hoc Tukey test for ANOVA, and its output consists of a critical difference (CD) threshold. In order to do that, ranks are assigned to algorithms. For each data set, the algorithm with the best performance gets the lowest (best) average rank. The mean performance of two despiking algorithms is judged to be signifycantly different if the corresponding average ranks differ by at least the critical difference (the graphical output of Nemenyi test was implemented using tools provided in the R package tsutils (https://CRAN.R-project.org/package=tsutils)).

## Results and discussion

### Modelling raw, high-frequency EC data

**Figure 2:**
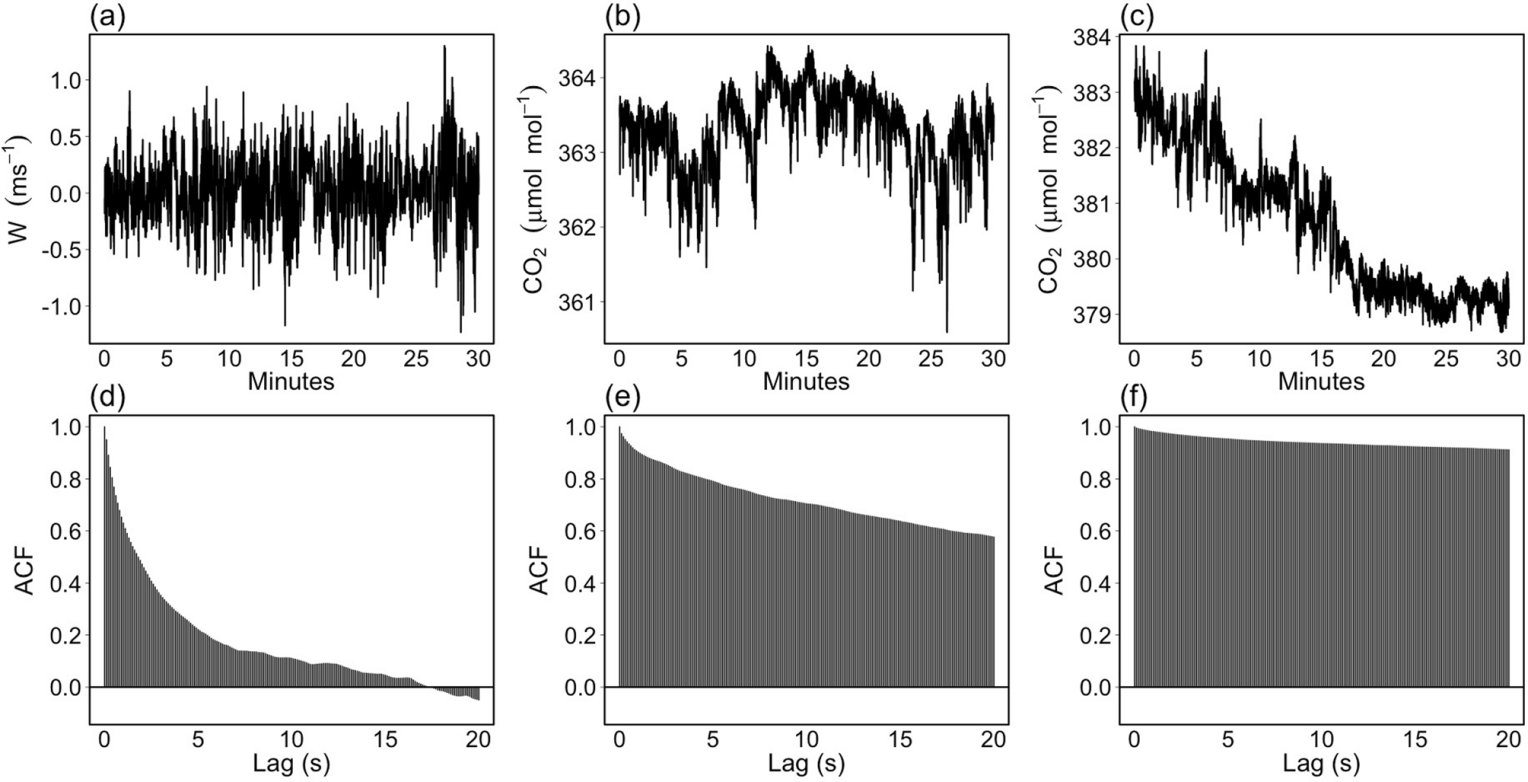
Raw, high-frequency vertical wind speed (*w*, m s$^{-1}$, **a**) and two time series of carbon dioxide concentrations (CO$_2$, $\upmu$mol mol$^{-1}$, **b, c**) from FI-Sii site. Their corresponding autocorrelation functions (ACF) are depicted in (**d**–**f**).

In the following, we report an illustrative example of the data modeling procedure carried out by means of the ARIMA-CGARCH model described in the “Monte Carlo experiment” section. To this end, data from FI-Sii site^29^ (Siikaneva, Finland, Boreal peatland) that is part of the Integrated Carbon Observation System - Research Infrastructure (ICOS-RI, https://www.icos-ri.eu) were used, and in particular, raw, high-frequency EC data of vertical wind speed (*w*, m s$^{-1}$) and carbon dioxide concentrations (CO$_2$, $\upmu$mol mol$^{-1}$) depicted in Fig. 2a–c.

**Figure 3:**
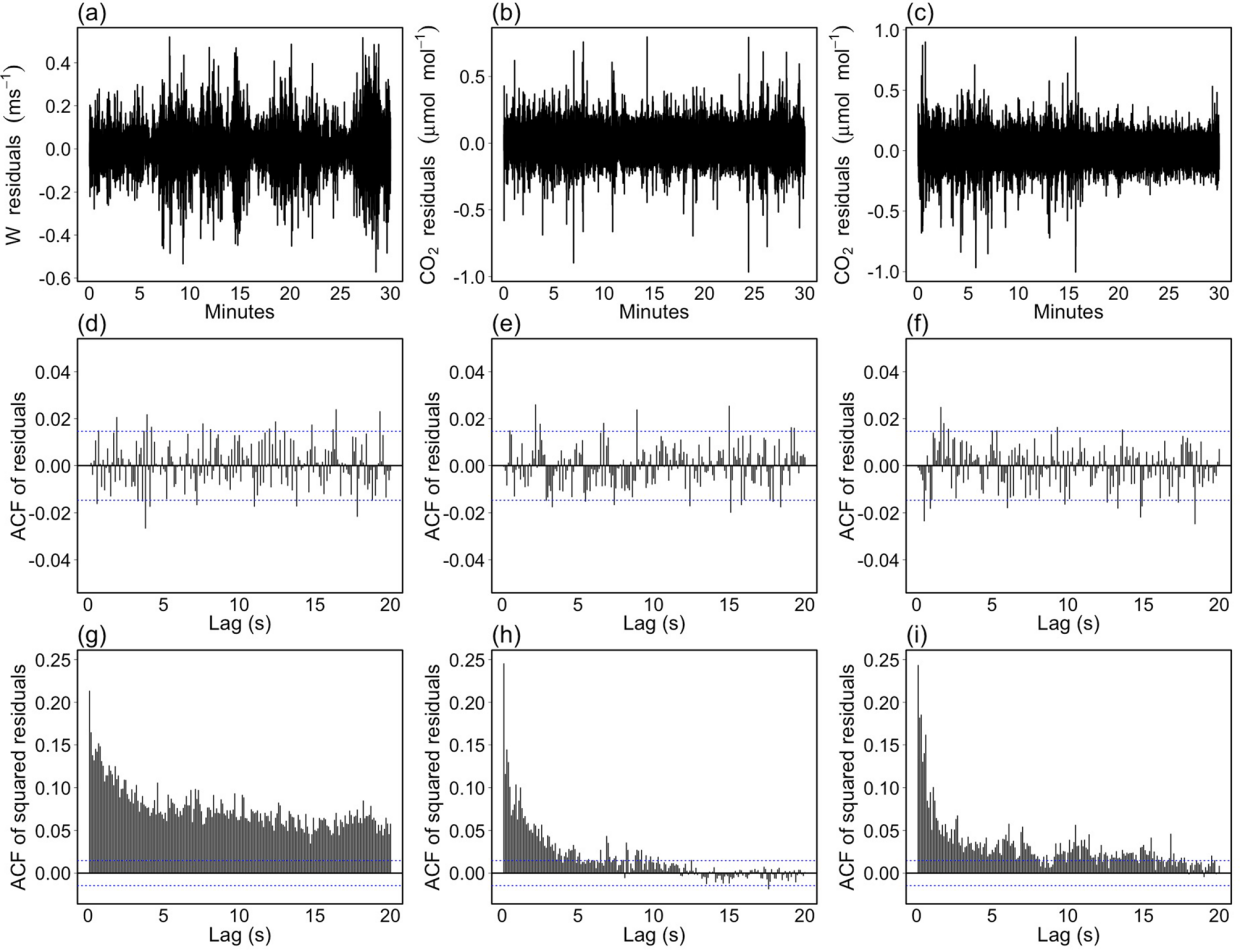
Diagnostic check of ARIMA models estimated on high-frequency time series depicted in Fig. 2. (**a**–**c**) Model residuals. (**d**–**f**) The corresponding autocorrelation functions (ACF). (**g**–**i**) The ACFs estimated on squared residuals. Dashed blue lines indicate the 95% CI.

**Figure 4:**
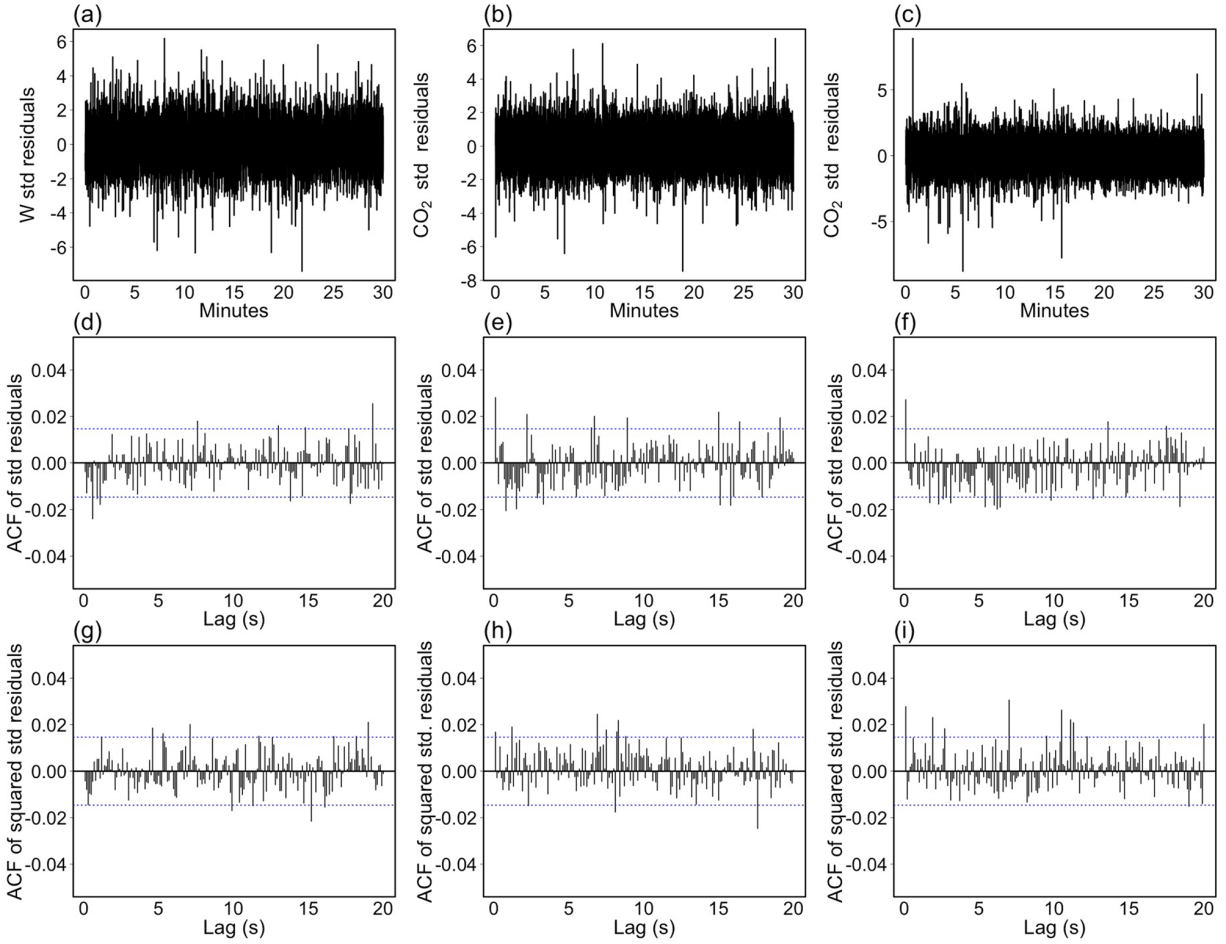
Diagnostic check of ARIMA-CGARCH models estimated on high-frequency time series depicted in Fig. 2. (**a**–**c**) The standardized residuals. (**d**–**f**) The corresponding autocorrelation function (ACF). (**g**–**i**) The ACFs estimated on squared standardized residuals. Dashed blue lines indicate the 95% CI.

High-frequency EC time series exhibit a high degree of serial correlation. The autocorrelation function (ACF) of *w* (Fig. 2d) decreases exponentially towards 0 as the time lag increase, while those of CO$_2$ (Fig. 2e,f) decay to 0 much more slowly. This behavior indicates that past values have a long-lasting impact on subsequent values and that high-frequency EC time series are highly persistent. A slow decaying of the ACF can also be caused by non-stationary conditions due to the presence of deterministic and/or stochastic trends as in the case of CO$_2$ time series depicted in Fig. 2c. This is confirmed by the non parametric Breitung variance ratio unit root test^30^ which led to the rejection of the null hypothesis of non-stationarity for *w* and the first CO$_2$ time series (p-value equal to 0.001 and 0.033, respectively), whereas indicated the presence of a stochastic trend in the second CO$_2$ time series (p-value=0.21 after linear trend removal). Therefore the order of integration *d* for the ARIMA specification was set equal to zero for vertical wind speed and the first, stationary CO$_2$ time series, whereas equal to one for the second, nonstationary CO$_2$ time series.

Among a set of ARIMA specifications, the most parsimonious model selected by means of the Akaike Information Criterion (AIC) resulted an ARIMA(4,0,1) for *w* and the stationary CO$_2$ time series and an ARIMA(5,1,1) for the non-stationary CO$_2$ time series.

The appropriateness of the selected models was inspected by examining the properties of the residuals. A well-specified model requires the residuals be uncorrelated. This implies that the serial correlation structure underlying observed data has been captured by the model and that the residuals are representative of the only (random) measurement error process. It is well known that for random and independent series of length *n*, the lag *k* autocorrelation coefficient is normally distributed with a mean of zero and a variance of 1/*n*, and the 95% confidence limits are given by $\pm 1.96/\sqrt{n}$. The ACFs depicted in Fig. 3d–f show that there is no significant autocorrelation left in the residuals from ARIMA-type models (autocorrelations at any lag of small magnitude can be considered negligible). However, while they seem statistically uncorrelated, the variability of residuals is not constant over time (Fig. 3a–c). Consequently, residuals cannot be considered identically distributed. Moreover, there is a tendency to the so-called volatility clustering where large (small) absolute values are followed by other large (small) values of unpredictable sign. Such behavior is strictly related to changes in turbulence regimes affecting most of the variables measured by EC systems. A further confirmation is provided by inspecting the ACFs of the squared residuals (Fig. 3g–i), which show significant correlations even at high lags, in particular for *w* time series.

To model such dynamics, we enriched the ARIMA specification with a CGARCH error structure. In particular, a CGARCH(1,1) specification was considered appropriate for the purpose of modeling the conditional variance process. The estimated parameters of the ARIMA-CGARCH models for *w* and CO$_2$ time series are reported in Table 2. Although the presence of unit root has been rejected, stationary *w* and CO$_2$ time series are characterized by a high degree of persistence of the conditional mean process (the sum of AR coefficients $\phi _i$ is close to one). At the same time, the persistence of the conditional variance process resulted higher for *w* (transitory and permanent components equal to 0.869 and 0.999, respectively) than for stationary CO$_2$ (transitory and permanent components equal to 0.231 and 0.962, respectively) and non-stationary CO$_2$ (transitory and permanent components equal to 0.730 and 0.988, respectively).

As before, the appropriateness of the selected models was assessed by inspecting the characteristics of model residuals. In particular, the ability of the ARIMA-CGARCH model in modeling the conditional mean and conditional variance processes underlying observed data, was evaluated by examining the presence of significant autocorrelations in both the residuals and the squared residuals standardized by the conditional standard deviation estimated by the CGARCH model. The standardized residuals and their respective ACFs are plotted in Fig. 4. Compared with Fig. 3a–c, standardized residuals resulted more homogeneous, although some significant correlation at shorter lags of negligible magnitude (panels d-f) has been observed. Nevertheless, the ACFs of squared standardized residuals (panels g-i) do not exhibit any significant correlation structures, meaning that most of the dynamics of the conditional variance process underlying observed data was successfully captured by the CGARCH model.

**Table 2:** ARIMA-CGARCH model parameters estimated for vertical wind speed (*w*) and two time series of carbon dioxide concentration (CO$_2$).

Parameter	W		CO$_2$ (stationary)		CO$_2$ (non-stationary)	
	Estimate	Std error	Estimate	Std error	Estimate	Std error
d	0	–	0	–	1	
c	0.026687	0.015946	363.476099	0.000639	0.000031	0.000202
$\phi _1$	1.991416	0.000439	1.380950	0.000340	0.501431	0.012053
$\phi _2$	$-$ 1.237142	0.000533	$-$ 0.239578	0.000539	0.115751	0.009213
$\phi _3$	0.303721	0.001093	$-$ 0.081151	0.003391	0.054348	0.009251
$\phi _4$	$-$ 0.061361	0.001303	$-$ 0.061146	0.003160	0.040066	0.009247
$\phi _5$	–	–	–	–	0.017344	0.007883
$\theta _1$	$-$ 0.861388	0.004251	$-$ 0.850201	0.004369	$-$ 0.913005	0.000973
$\omega$	0.000015	0.000002	0.000456	0.000058	0.000139	0.000007
$\alpha _1$	0.106533	0.007681	0.107306	0.012461	0.127860	0.000912
$\beta _1$	0.762403	0.028287	0.123850	0.098535	0.602410	0.029983
$\eta _{11}$	0.998971	0.000029	0.962593	0.004987	0.988055	0.000026
$\eta _{21}$	0.049175	0.002766	0.071236	0.006574	0.024012	0.001645

### Performance evaluation

**Figure 5:**
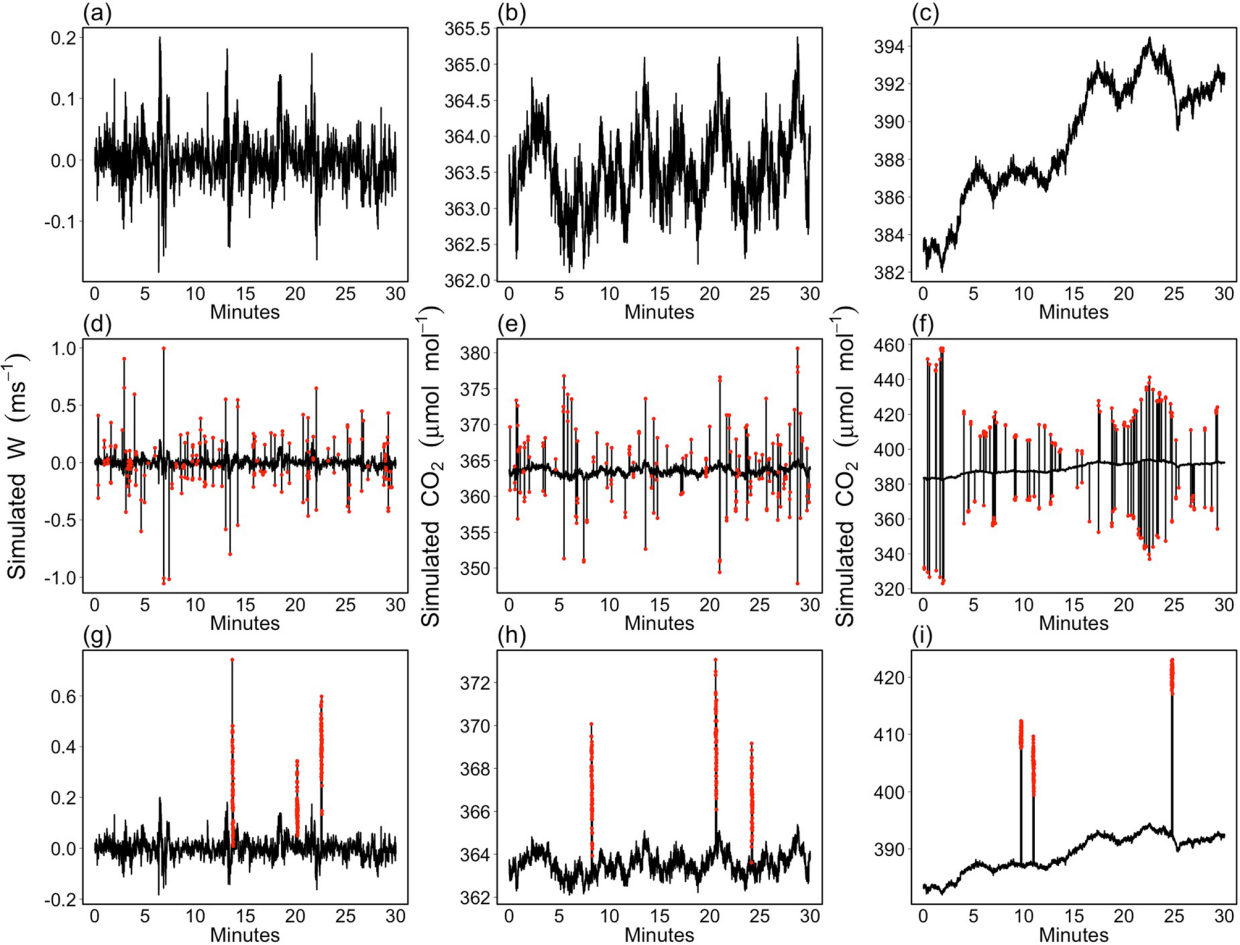
Illustrative examples of simulated time series of vertical wind speed (*w*, m s$^{-1}$, **a**) and stationary (**b**) and non-stationary (**c**) carbon dioxide concentration (CO$_2$, $\upmu$mol mol$^{-1}$). (**d**–**f**) and (**g**–**i**) The same time series corrupted with spike events (red dots) according to S1 and S2 scenarios, respectively.

In the light of the considerations reported in the previous section, data were simulated from three ARIMA-CGARCH model specifications following the parameter settings reported in Table 2. The first simulation model (hereinafter denoted as M$_W$) aims at mimicking the high persistence of the conditional variance process as often observed in the wind velocity components. The other two simulation models aim at mimicking the high persistence of the conditional mean process, as often observed in the scalar concentration variables. In particular, one of them (hereinafter denoted as M$_{C}$) simulates stationary highly persistent time series, whereas the other (hereinafter denoted as M$_{C_T}$) simulates non-stationary time series affected by the presence of a stochastic trend. Simulated data from the three ARIMA-CGARCH model specifications were then corrupted with spike events according to the S1 and S2 scenarios described in the “Monte Carlo experiment” section. Illustrative examples of simulated time series are depicted in Fig. 5.

**Figure 6:**
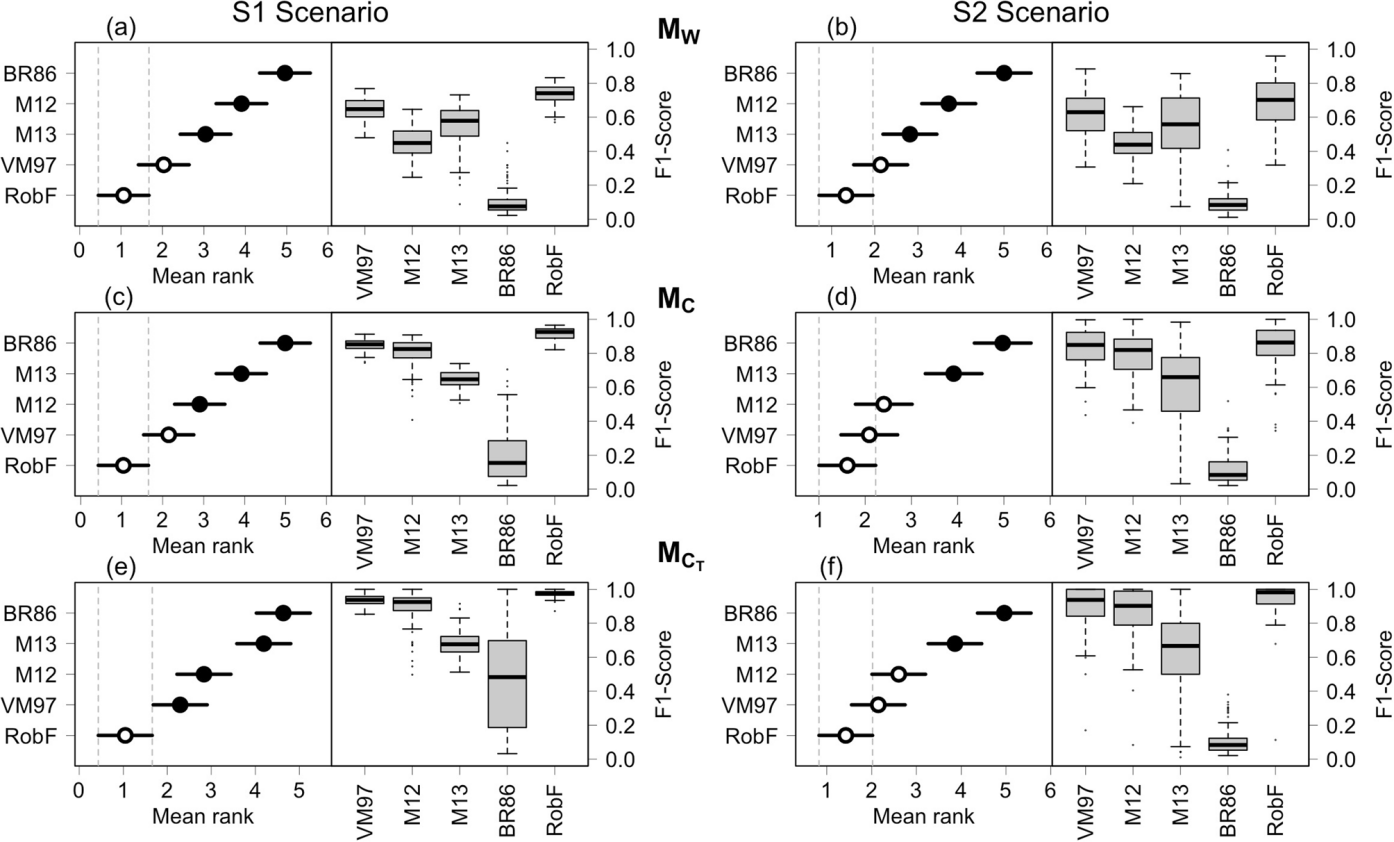
Graphical visualization of the Nemenyi test for the evaluation of the F1-Score performance metric. The algorithm with the lowest rank is the best performing. Panels a and b show the results obtained in S1 and S2 scenarios, respectively, using data simulated from the M$_W$ model. Panels c and d r show the results obtained in S1 and S2 scenarios, respectively, using data simulated from the M$_C$ model. Panels e and f show the results obtained in S1 and S2 scenarios, respectively, using data simulated from the M$_{C_T}$ model. White dots indicate there is not statistically significant difference between the performances of the algorithms. In all scenarios, the null hypothesis of the Friedman test that there are no significant differences between performances of all the considered algorithms is rejected at 0.05% significance level.

Table 3 reports Precision, Recall and F1-Score metrics used for performance evaluation of the despiking algorithms. To aid in comparison, the results of Monte Carlo simulations were summarized through standard box-plots and Nemenyi Critical Difference (CD) plots, separately for simulation model (M$_W$, M$_C$, M$_{C_T}$) and S1 and S2 scenarios. The comparison of F1-Score metrics calculated for each despiking algorithm is summarized in Fig. 6.

On average, no significant difference was observed between performances achieved in S1 and the ones in S2, meaning that the ability of each algorithm in detecting spiky events mainly depends on the spike magnitude rather than on their location. Compared to the S1 scenario, performance metrics for S2 showed higher variability for most of the algorithms. Consecutive spikes, in particular when they follow a curvilinear pattern, resulted more difficult to detect, in particular by methods that assume continuity of data distribution (e.g. BR86). Significantly differences in terms of performances were observed in relation to the stochastic properties simulated by the ARIMA-CGARCH models.

Concerning the S1-M$_C$ and S2-M$_C$ scenarios with simulated data characterized by a high degree of persistence of the (stationary) conditional mean process, good performances were reached by the VM97, M12 and RobF procedures (F1-Score> 0.75). The latter resulted the best performing (F1-Score equal to 0.91 and 0.84 in S1 and S2 scenarios, respectively). A medium to low performance was observed for the M13 approach (F1-Score equal to 0.64 and 0.62 in the S1-M$_C$ and S2-M$_C$ scenarios, respectively), whereas a poor performance was observed for the BR86 procedure (F1-Score equal to 0.20 and 0.12 in S1-M$_C$ and S2-M$_C$ scenarios, respectively) algorithm. Although excellent in terms of Precisions, the method suffers in terms of Recall (< 0.4 in all the simulated scenarios). By construction, in fact, BR86 is unable to detect spikes when they occur in different size and magnitude because the empty bins in the histogram of the differences between raw signal and median filtered signal separate only the biggest spikes (this deficiency could be probably alleviated by using more bins than 25 already in the beginning of the despiking procedure). As a consequence, spiky events remain undetected although falling into the tails of the distribution.

Similar considerations were found for the S1-M$_{C_T}$ and S2-M$_{C_T}$ scenarios. In particular, it was found a greater variability of results for M13 in the S2-M$_{C_T}$ scenario. More likely M13 suffers when time series exhibit pronounced time trend dynamics and consecutive spikes remains undetected due to the intrinsic larger variability of data.

In the case of simulated data characterized by a high degree of persistence of the conditional variance process (M$_W$ ARIMA-CGARCH model parameter setting), the performance of each despiking algorithm was subject to a significant deterioration. The reason is that the higher persistence of the conditional variance process introduced by the M$_W$ model specification makes more difficult to distinguish between good data and spiky events. As a consequence a reduction of the Recall metric was observed for each despiking algorithm. Overall, compared with existing procedures a superior performance of the RobF approach was observed (F1-Score equal to 0.74 and 0.68 in S1-M$_W$ and S2-M$_W$ scenarios, respectively). Such a performance is mainly driven by the Precision metric (0.96 in both the S1-M$_W$ and S2-M$_W$ scenarios). VM97, M12 and M13 algorithms showed a medium to low performance both in terms of Precision and Recall. A poor performance was instead observed for the BR86 procedure (F1-Score equal to 0.10 and 0.11 in S1-M$_W$ and S2-M$_W$ scenarios, respectively) for the same reasons previously reported.

**Figure 7:**
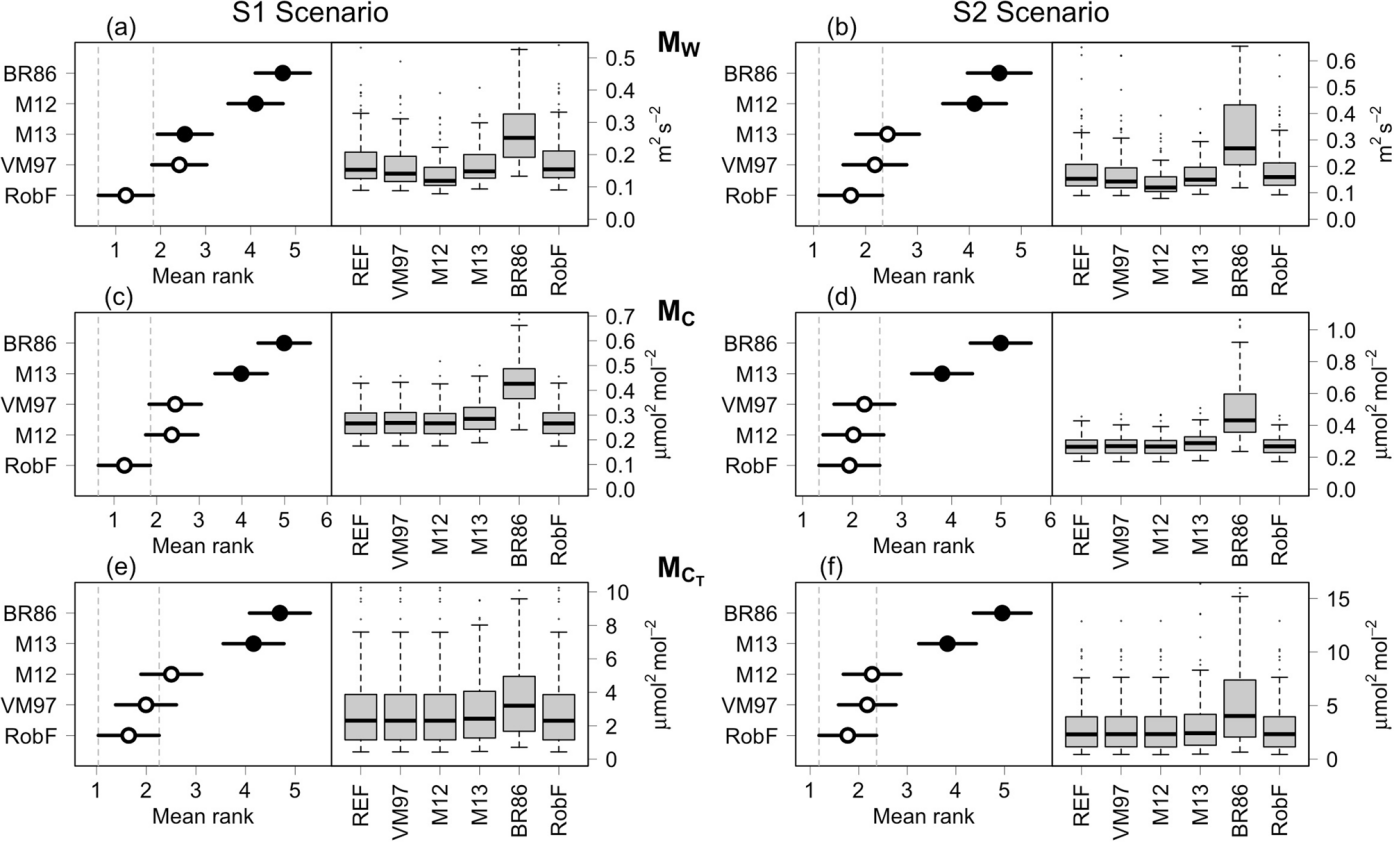
Graphical visualization of the Nemenyi test for the comparison of variance estimates of uncorrupted (REF) and despiked time series according to VM97, M12, M13, BR86 and RobF algorithms. The algorithm with the lowest rank is the best performing, meaning that the difference between variances is the lowest in absolute terms. Panels a and b show the results obtained in S1 and S2 scenarios, respectively, using data simulated from the M$_W$ model. Panels c and d show the results obtained in S1 and S2 scenarios, respectively, using data simulated from the M$_C$ model. Panels e and f show the results obtained in S1 and S2 scenarios, respectively, using data simulated from the M$_{C_T}$ model. White dots indicate there is not statistically significant difference between the results achieved by the despiking algorithms. In all scenarios, the null hypothesis of the Friedman test that there are no significant differences between performances of all the considered algorithms is rejected at 0.05% significance level.

Failure to detect spiky events can introduce significant biases in variance estimation and, consequently, in turbulence statistics involved in flux calculation. To this end, we compared the variance of uncorrupted (simulated) time series and the one estimated after the despiking procedure. Figure 7 summarized the results through standard box-plots and Nemenyi Critical Difference (CD) plots, separately for simulation model (M$_W$, M$_C$, M$_{C_T}$) and S1 and S2 scenarios. The best performing algorithm in recovering the variance of time series was RobF, followed by VM97 and M12. M13 showed a good performance in the S1-$M_W$ and S2-$M_W$ scenarios, whereas the variance of despiked time series by means of BR86 method resulted significantly higher, a symptom that large spikes remained likely undetected.

Concerning the time required to run the different despiking procedure, M13 and BR86 resulted the most computationally efficiency algorithms (CPU time less than 0.1 s for each run using a 2.2 GHz Intel Core i7 CPU). The time required for the RobF procedure ranges from 1 to 3 s, depending on the window width selected for the RM filter. The computational cost for VM97 and M12 resulted more relevant due to the iterative procedure involved in spike detection (5–10 s for each run).

**Table 3:** Performance evaluation metrics of despiking methods.

Simulation	Despiking	S1 scenario			S2 scenario		
Model	Algorithm	Precision	Recall	F1-Score	Precision	Recall	F1-Score
M$_{W}$	VM97	0.64	0.66	0.65	0.63	0.61	0.61
	M12	0.34	0.73	0.45	0.34	0.72	0.45
	M13	0.72	0.48	0.55	0.71	0.48	0.54
	BR86	1.00	0.06	0.10	0.93	0.05	0.10
	RobF	0.96	0.60	**0.74**	0.96	0.54	**0.68**
M$_{C}$	VM97	0.99	0.74	0.85	0.98	0.73	0.83
	M12	0.87	0.75	0.80	0.87	0.75	0.79
	M13	1.00	0.48	0.64	1.00	0.48	0.62
	BR86	1.00	0.12	0.20	1.00	0.06	0.12
	RobF	1.00	0.85	**0.91**	0.99	0.75	**0.84**
M$_{C_T}$	VM97	0.99	0.88	0.93	0.99	0.84	0.90
	M12	0.92	0.88	0.89	0.91	0.86	0.86
	M13	1.00	0.53	0.69	1.00	0.51	0.63
	BR86	1.00	0.36	0.47	1.00	0.06	0.11
	RobF	1.00	0.95	**0.97**	1.00	0.91	**0.94**

The highest F-Score metric (i.e., best perfomance) for each scenario is shown in bold.

### Application to EC data

In the following, an evaluation of the ability of the despiking algorithms in spike removal on real EC data is provided.

To appreciate the disturbing effect caused by spikes, we compared the cross-covariance functions (CCF) between *w* and CO$_2$ time series sampled in two consecutive blocks of 30-min time intervals characterized by similar meteorological conditions and where similar CO$_2$ fluxes in magnitude are expected.

**Figure 8:**
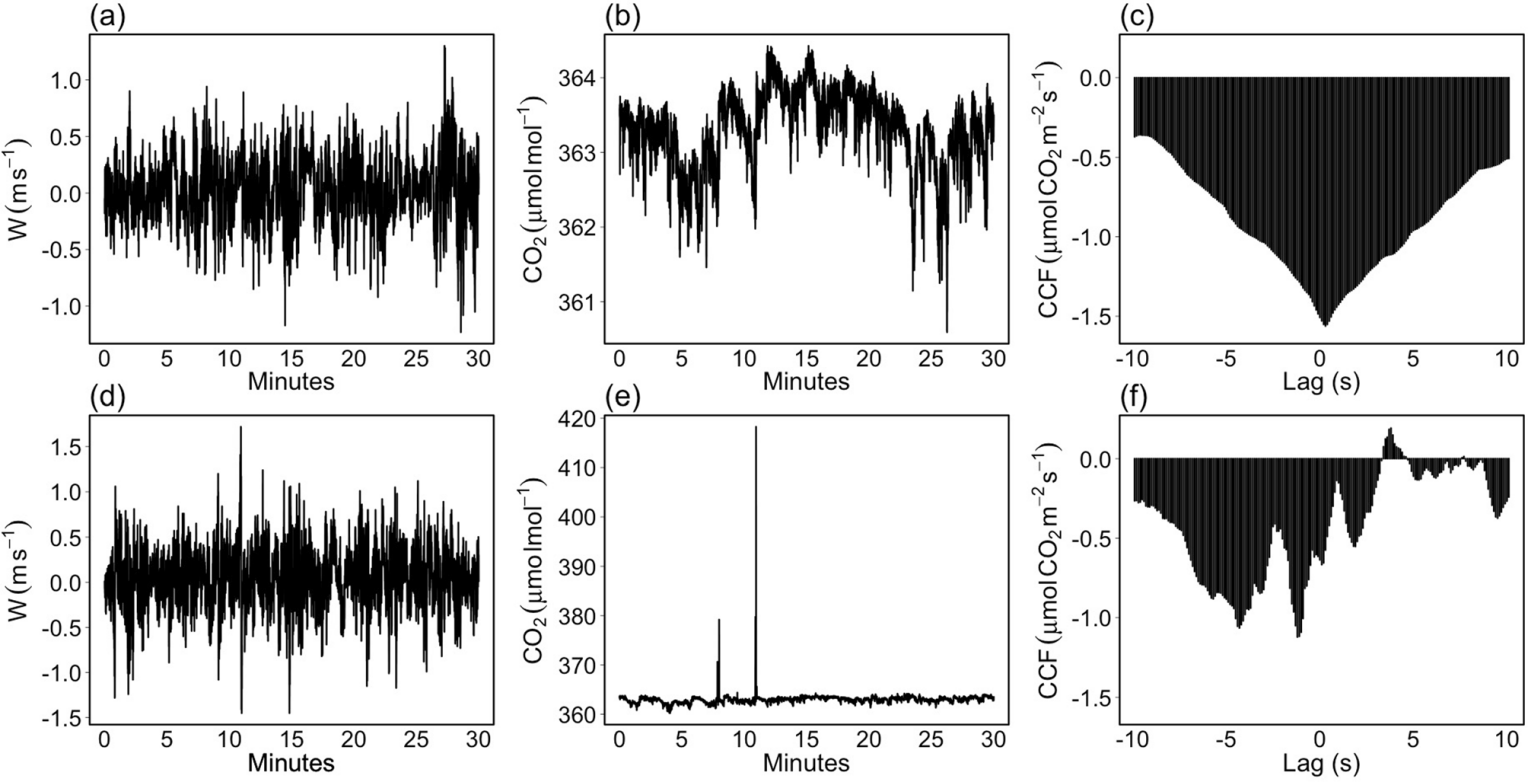
Raw, high-frequency vertical wind speed (*w*, m s$^{-1}$, **a, d**), carbon dioxide concentration (CO$_2$, $\upmu$mol mol$^{-1}$, **b, c**) time series and their cross-covariance function (CCF, $\upmu$mol CO$_2$ m$^{-2}$ s$^{-1}$) sampled at FI-Sii site on 2017-07-25 in two subsequent blocks of 30 min (17:00–17:30 **a**–**c**; 16:30–17:00, **d**–**f**). The shape of the CCF depicted on (**c**), and characterized by a well-defined peak, represents an ideal case for EC data.

A careful visual inspection revealed that the time series sampled in the last 30 min (Fig. 8a, b) were not affected by spikes (note that time series are the same depicted in Fig. 2a,b). The shape of the CCF (Fig. 8c) exhibits a dominant peak at time lag 0.3 s with a flux estimate equal to $-1.5\,\upmu$mol CO$_2$ m$^{-2}$ s$^{-1}$ (such a delay is caused by the sample transit time through the sampling line of the gas analyzer; once the time lag has been detected, the time series can be synchronized, and the flux value is given by the covariance derived from the matched time series).

The CO$_2$ time series sampled in the first 30 min (Fig. 8e) shows instead evident impulsive peaks consisting of a sequence of more than 30 consecutive anomalous values. Such contaminations are responsible to introduce significant biases in the CCF estimates. In particular, the dominant peak disappears (Fig. 8f) and the estimated flux at the time lag 0.3 s is around $-0.5\,\upmu$mol CO$_2$ m$^{-2}$ s$^{-1}$, a quantity 70% lower than those estimated in the subsequent 30-min interval.

In such a case, an effective despiking procedure is essential to prevent biased flux estimates. We therefore performed the despiking algorithms described in the “Methods” section. In this application, the maximum number of consecutive spikes allowed by VM97 and M12 was set equal to 4, as recommended by Vickers and Mahrt^6^.

**Figure 9:**
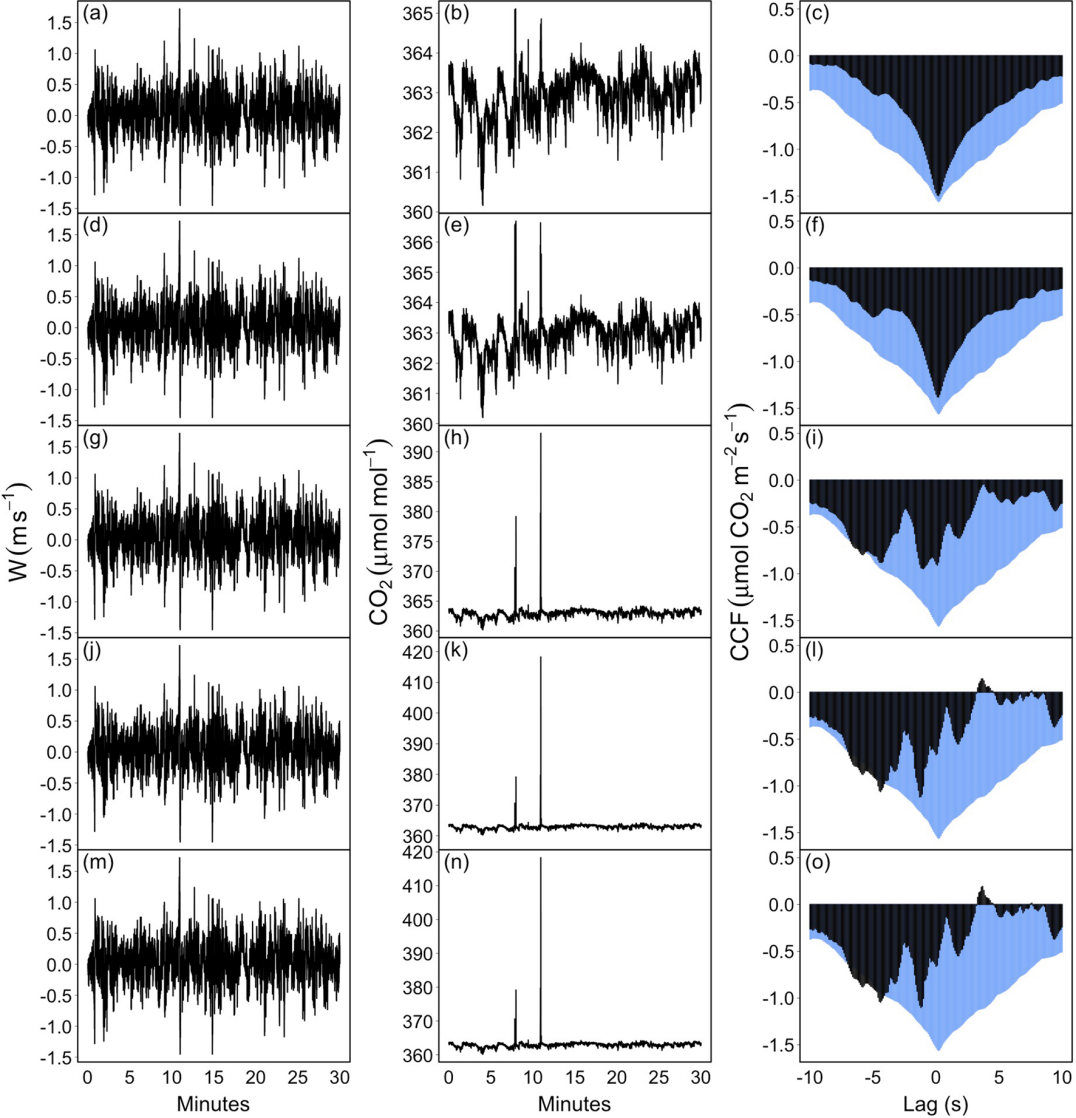
An application of despiking algorithms on real EC data from FI-Sii site: vertical wind speed (*w*, m s$^{-1}$, left panels), carbon dioxide concentration (CO$_2$, $\upmu$mol mol$^{-1}$, middle panels), and their cross-covariance function (CCF, $\upmu$mol CO$_2$ m$^{-2}$ s$^{-1}$, right panels). (**a**–**c**) The despiked time series and their CCF after application of the RobF procedure. (**d**–**f**) The despiked time series and their CCF after application of the M13 algorithm. (**g**–**i**) The despiked time series and their CCF after application of the BR86 algorithm. (**j**–**l**) The despiked time series and their CCF after application of the VM97 algorithm. (**m**–**o**) The despiked time series and their CCF after application of the M12 algorithm. Original raw data are depicted in Fig. 8d,e. The blue colored CCF on the right panels refers to the one estimated in the subsequent 30 min and is used as a reference (see Fig. 8c).

After the removal of the impulsive peaks in CO$_2$ time series by the RobF procedure (Fig. 9b), the CCF returns to its ideal shape (Fig. 9c), with a flux estimate in correspondence of the dominant peak of about $-1.5\,\upmu$mol CO$_2$ m$^{-2}$ s$^{-1}$, a quantity consistent with those estimated in the subsequent 30 min. A significant improvement of the shape of the CCF was also achieved by the M13 despiking algorithm (Fig. 9f). However, a number of undetected spikes remain in the CO$_2$ time series (Fig. 9e). As a consequence flux covariance estimation resulted lower of about 8% than those estimated with despiked data by the RobF procedure. The other algorithms were not able to detect and remove spikes. The BR86 algorithm removed only the largest spikes in magnitude (Fig. 9h), a removal nevertheless not sufficient to recover the ideal shape of the CCF (Fig. 9i) and achieve unbiased flux estimate. Limiting the spike events to a short duration did not allow the VM97 and M12 procedures to remove the spurious peaks (Fig. 9k,n, respectively). To increase their effectiveness, a higher number of consecutive spikes should be specified, although this could imply longer computational times due to iterations involved.

## Conclusions

The eddy-covariance (EC) technique is considered the most direct and reliable method to calculate flux exchanges of the main greenhouse gases over natural ecosystems and agricultural fields. The resulting measurements are extremely important to characterize ecosystem exchanges of carbon, water, energy and other trace gases, and are widely used to validate or constrain process-based models.

Despite improvements in measurement instruments and data acquisition systems, rigorous quality control (QC) procedures are required to prevent biased flux estimates. One of the QC routines deals with the identification of unexpectedly impulsive peaks (spikes) contaminating EC raw data. A variety of procedures have been proposed for the detection and removal of spikes. However, there is no general agreement as to which method is the best, because the performance of the despiking algorithms for EC data is generally unknown.

Spike detection for raw, high-frequency EC time series is a challenging task because of the confounding effect caused by complex dynamics and the high level of noise affecting such data. To cope with these features, a new despiking procedure rooted on robust functionals is proposed. The procedure involves a preliminary signal extraction carried out by means of the repeated median regression technique^10,17^. Spikes are detected through the examination of outlier scores obtained by scaling the residuals with a robust time-varying estimate of the scale parameter, based on the $Q_n$ estimator^12^.

The performance of the proposed approach was evaluated by means of a Monte Carlo experiment. To aid in comparison, four established methods for spike detection in high-frequency EC data were considered. Specifically, we considered two moving time-window based approaches (VM97, M12), an approach based on global estimates (M13), and a procedure based on a nonlinear median filter with threshold logic (BR86).

Time series were simulated from three ARIMA-CGARCH models with the aim to mimic the main stochastic properties underlying observed EC data as closely as possible. Subsequently, simulated data were corrupted with spiky events of size similar to those commonly encountered in real, observed data. Although the simulation design covers only a limited range of among cases observed in the experimental measurements, such an approach allows a full control of time series dynamics and, consequently, a more objective performance evaluation of the despiking methods.

Within this simulation framework, it is demonstrated that the proposed procedure performs better than the existing algorithms and can be therefore considered as a candidate for the implementation in data center environmental monitoring systems, where the availability of automatic procedures ensuring a high quality standard of released products constitutes an essential prerequisite.

## Data Availability

EC data used^31,32^ are from the Integrated Carbon Observation System (ICOS) European Research Infrastructure and accessible through the ICOS Carbon Portal (www.icos-cp.eu). The proposed RobF despiking procedure is implemented in the R package RFlux^33^ and available at https://github.com/icos-etc/RFlux.
